# Unraveling the assembly mechanisms and differentiated ecological functions of protist cell-associated and free-living bacterial communities during two *Prorocentrum shikokuense* blooms

**DOI:** 10.1128/spectrum.02451-24

**Published:** 2025-05-15

**Authors:** Xiaoyu Wang, Xin Lin, Huatao Yuan, Hao Luo, Sitong Lin, Wenxue Wu, Ling Li

**Affiliations:** 1 State Key Laboratory of Marine Environmental Science, College of Ocean and Earth Sciences, Xiamen University 12466, Xiamen, Fujian, China; 2 State Key Laboratory of Marine Resource Utilization in South China Sea, Hainan University 74629, Haikou, China; Connecticut Agricultural Experiment Station, New Haven, Connecticut, USA

**Keywords:** Dinoflagellate bloom, *Prorocentrum shikokuense*, phycosphere, bacterial community diversity, community assembly mechanism

## Abstract

Free-living and cell-associated microbial communities are crucial in the development, maintenance, and decline of harmful algal blooms (HABs), yet little is known about how they differ and change during bloom succession and whether the phycosphere-associated bacterial community of an algal species remains similar in different geographic populations. This study employed 16S rRNA gene amplicon sequencing on size-fractionated samples to investigate community assembly, dynamics, and co-occurrence patterns of free-living and cell-associated bacterial communities during two *Prorocentrum shikokuense* (Dinophyceae) blooms in the East China Sea. The two communities exhibited distinct compositions and dynamics changes, influenced by varying degrees of assembly processes and environmental factors. Core phyla—Proteobacteria (25.8%–57.1% in cell-associated communities, 37.3%–55.5% in free-living communities), Bacteroidota (12.5%–25.1%; 13.3%–18.5%), Actinobacteriota (6.7%–16.1%; 23.7%–33.3%), and Planctomycetota (3.6%–40.7%; 2.4%–5.6%)—comprised 81.8%–95.8% of observed amplicon sequence variants. The cell-associated community structure changed more evidently during bloom development compared to free-living communities, sharing a similar structure despite spatial and temporal differences. Environmental factors contributed more to the variance in free-living communities. Homogeneous selection predominantly shaped free-living composition, while cell-associated communities were more influenced by stochastic processes, especially dispersal limitation. Cell-associated community diversity was determined by residence space rather than the geographic location or bloom stage, with simpler co-occurrence networks compared with free-living communities. Furthermore, cell-associated communities showed enriched ecological functions in nutrient cycling and cytolysis. The ecological implications of the higher abundance of Rhizobiales in cell-associated communities for nitrogen fixation were also discussed. This work enhances our understanding of algal-bacteria interactions and the distinct dynamics between free-living and cell-associated communities.

**IMPORTANCE:**

Microbial communities are crucial in the development of harmful algal blooms (HABs), yet how free-living and cell-associated bacterial communities differ and change during bloom succession remains unclear. Using 16S rRNA gene amplicon sequencing, we investigated the dynamics and assembly mechanisms of these two community types during two *Prorocentrum shikokuense* blooms in the East China Sea. Our findings reveal distinct structural compositions and dynamics between cell-associated and free-living communities, shaped by varying assembly processes and environmental factors. Cell-associated bacteria in the phycosphere of *P. shikokuense*, strongly influenced by dispersal limitation due to their close interaction with the algal host, exhibit enriched functions in nutrient cycling and cell lysis. This suggests that cell-associated bacteria may play an essential role in algal bloom development and dissipation. This research broadens our understanding of algae-bacteria interactions and microbial community dynamics during harmful algal blooms, offering valuable information for managing algal blooms and protecting marine ecosystems.

## INTRODUCTION

Harmful algal blooms (HABs) are characterized by increased biomass and rapid proliferation of bloom-forming species, causing damage to the aquatic ecosystem and even human health (1, 2). In recent years, the frequency, duration, and magnitude of HAB events have increased significantly due to eutrophication (3–5). Environmental conditions and interactions between different species jointly affect microalgae growth, thus regulating the algal blooms (6). A study on the succession of phytoplankton and microbial communities following a bloom off southern California found that phytoplankton and prokaryotic communities are more strongly correlated to each other than to environmental parameters, based on Mantel test analysis (7). Some studies also suggest that interactions between phytoplankton and prokaryotic communities are crucial to the dynamic changes of phytoplankton communities (8–11).

The phycosphere, a microscale zone surrounding a phytoplankton cell, is rich in extracellular metabolites released by the cell that attract a large number of bacteria (12, 13). Cell-associated bacteria (CA), which bind to algal cells, exhibit significant differences in community structure and functional metabolism from the free-living bacteria (FL) in the surrounding seawater (14, 15). CA bacteria are believed to be intimately associated with algae and can promote or inhibit algal growth during algal blooms (8). Algae provide the bacteria with fixed organic carbon, and the bacteria, in return, supply nutrients such as nitrogen, phosphorus, and vitamin B12, which are necessary for algae growth (16). Meanwhile, bacteria can also pose a threat to algae by competing with them for existing nutrients or by degrading their cell walls (17). The interaction between algae and bacteria could be species-specific, and different growth and physiological statuses will lead to a change in the bacterial community structure (17–19). A case study on the *Microcystis* phycosphere revealed that large (>100 µm)- to small (0.2–10 μm)-sized cyanobacterial cell-associated bacteria can degrade high- to low-molecular-weight compounds to promote efficient recycling of nutrients (20). In a *Gymnodinium*-diatom bloom, the phycosphere bacterial community succession was affected by algal species instead of environmental factors (21).

Stochastic and deterministic processes are two principal mechanisms in driving the assembly of microbial communities (22). Stochastic processes involve birth, death, colonization, extinction, and speciation, which account for the randomness in the patterns of species composition (23, 24). Deterministic processes mainly include ecological selection driven by both biotic and abiotic factors such as species characteristics, interspecies interactions, and environmental conditions (25–27). A 4-year continuous study on the successions of bacterial communities during algal blooms in the southern North Sea revealed that these successions might be dominated by deterministic processes such as substrate-induced forcing, where specific substrates produced by algae drive the succession of bacterioplankton composition (28). Assembly processes of microbial communities in a research on dinoflagellate *Scrippsiella acuminata* (formerly *S. trochoidea*) bloom indicated that the structure of bacterioplankton communities correlates with bloom progression, and both CA and FL microbial successions are influenced by a combination of deterministic and stochastic processes (29). Deciphering the assembly mechanism that regulates both microbial communities lays the foundation for further addressing the alga-bacteria relationship and ecological effects during bloom events (30, 31).

*Prorocentrum shikokuense* is a widely distributed bloom-forming dinoflagellate that significantly impacts marine ecosystems. Although the species is nontoxic, its blooms can lead to substantial ecological disruptions, particularly in adjacent waters of the East China Sea (ECS) (32–34), as well as in Japanese and Korean coastal waters (35). Research indicated that the *P. shikokuense* blooms lead to decreased population growth, reduced egg production rates, and increased mortality in various zooplankton (36–39), as well as decreased gross conversion efficiencies (calculated as the dry weight increment of the species divided by the dry weight of algae grazed by the species) and reduced larval survival rate in aquaculture species such as *Artemia salina* (40) and *Haliotis discus hannai* (41). In addition, *P. shikokuense* blooms can promote the growth of other harmful species, such as *Karlodinium veneficum* (42) and *Skeletonema costatum* (43), leading to shifts in algal community dynamics. Though the formation and development of *P. shikokuense* blooms have been extensively investigated (44), the diversity of FL and CA bacterial communities and the mechanism of community assembly in association with the bloom succession process remain limitedly explored.

In this study, metabarcoding based on the V3–V4 region of the 16S rRNA gene was employed to investigate the dynamic shift process and co-occurrence patterns of both FL and CA bacterial communities of two independent *P. shikokuense* bloom events in the ECS. The study aimed to address two major issues: (1) the composition differences and assembly mechanisms of FL and CA bacterioplankton community during the blooms and (2) whether *P. shikokuense* harbored a unique and resident bacteria community in their phycosphere. Additionally, the ecological role of bacteria during the development and dissipation of *P. shikokuense* blooms was discussed based on the predicted functions of the detected bacterial communities.

## MATERIALS AND METHODS

### Study sites and sample collection

**Fig 1:**
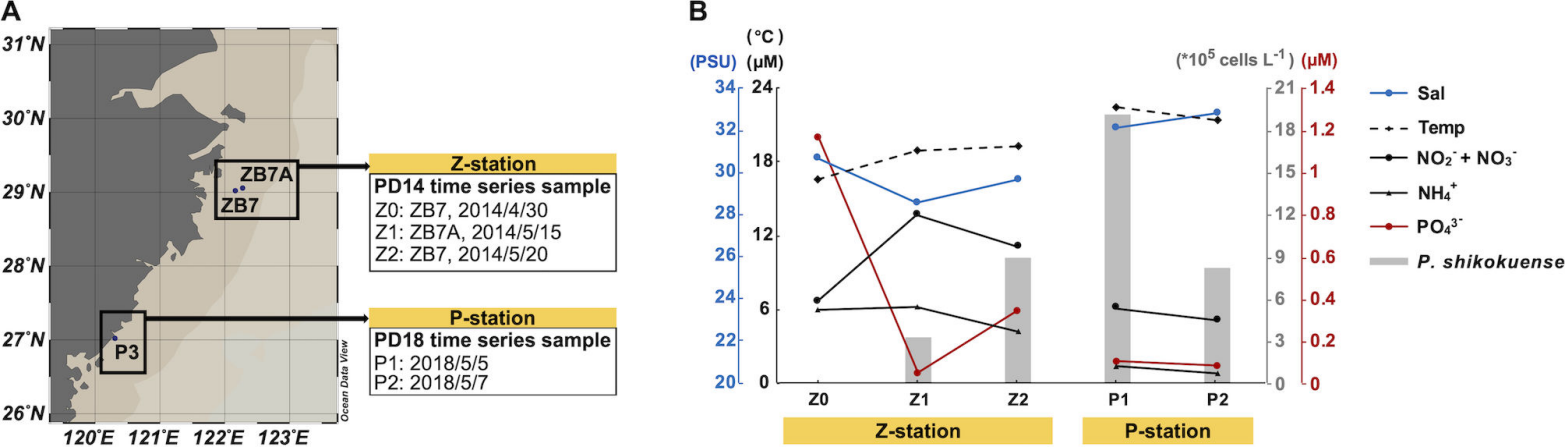
Geographic locations and environmental conditions of the sampling stations. (**A**) Sampling stations, Station ZB7 (29°1'0"N ;122°9'27"E) and ZB7A (29°3'19"N; 122°16'30"E) are located near the Yangtze River Estuary, ECS. Station P3 (27°1'17"N; 120°18'37"E) is located in the area of Sansha Bay; (**B**) Environmental conditions and *P. shikokuense* cell counts at the sampling stations. Environmental parameters include seawater temperature (Temp) and salinity (Sal), the concentrations of nitrate and nitrite (NO_2_^–^ + NO_3_^–^), ammonium (NH_4_^+^), and phosphate (PO_4_^3–^).

In this study, time-series samples of both FL and CA bacterioplankton were collected from two individual *P. shikokuense* bloom events in 2014 and 2018 in the ECS (Fig. 1A), PD14 and PD18 in abbreviation. According to our previous study of the PD14 event in the Changjiang estuary (45), sampling stations ZB7 and ZB7A, which are located 13 km apart, were treated as an integral area, where three-time series samples were collected on April 30 (Z0, non-blooming stage), May 15, and May 20 (Z1 and Z2, blooming stage), respectively (Fig. 1). The PD18 event occurred in Sansha Bay, a semi-enclosed bay in the northeastern area of Fujian, China. Time-series samples were collected at station P3 (27°1'16.8708"N; 120°18'36.6696"E) on May 5 and May 7, 2018 (namely, P1 and P2), representing the bloom peak and decline stage individually. The sampling map was plotted using the software Ocean Data View (46).

For each sample, 4L surface water (0–2 m) was first filtered through a 200 µm nylon mesh to remove zooplankton. The filtrate was then filtered through a 3 µm pore-size polycarbonate membrane, and the biomass retained on the membrane was used to extract algae cell-associated bacteria. The seawater filtered through a 3 µm pore-size filter was then passed again through a 0.2 µm polycarbonate membrane, and the cells on the membrane were treated as FL. Each membrane was immediately transferred into a microcentrifuge tube containing 1 mL DNA lysis buffer (0.1 M EDTA and 1% SDS) and stored at −80°C prior to DNA extraction, with each sample in triplicate. Meanwhile, 50 mL seawater samples were collected from each sampling station, fixed in Lugol’s solution with a final concentration of 2%, and stored in the dark for subsequent cell counting using a Sedgwick-Rafter counting chamber.

In the subsequent analysis, the CA and FL communities at the Z station during the bloom stage will be referred to as ZA and ZF, respectively (i.e., ZA is the CA samples of Z1 and Z2 and ZF is the FL samples of Z1 and Z2). Similarly, PA and PF will be used to represent the CA and FL samples of P1 and P2, respectively.

### DNA extraction, PCR amplification, and sequencing

DNA extraction was carried out following the bead-beating protocol previously described (47). The polycarbonate membrane was cut into pieces using sterilized scissors and then incubated in 500 µL of DNA lysis buffer and 10 µL of proteinase K for 48 hours at 56°C. The suspension was transferred to a new centrifuge tube, and approximately 0.05–0.15 g (equivalent to the cell pellet volume) of a 1:1 (vol/vol) mixture of 0.1 mm and 0.5 mm diameter ceramic beads was added. The samples containing beads underwent complete lysis using a FastPrep-24 bead mill (MP Biomedicals, USA) for bead-beating at a speed of 6 m/s for 45 seconds. This procedure was carried out three times, with 1-minute intervals between each repetition, while the samples were kept on ice. The supernatant was carefully transferred into a new tube, followed by the addition of 82.5 µL of 5M NaCl and 82.5 µL of 10% wt/vol CTAB, and incubation at 56°C for 10 minutes. Subsequently, an equal volume of chloroform (665 µL) was added to the sample, and the sample was vortexed until it became milky white. After centrifugation at ~13,523 × *g* for 10 minutes, the supernatant was transferred to a new tube. The subsequent purification procedure was performed using the DNA Clean & Concentrator kit (Zymo Research, USA), following the manufacturer’s protocol.

The 16S rRNA gene V3–V4 region (~466 bp) was amplified using the primers 314F: 5’CCTACGGGNGGCWGCAG and 806R: 5’GGACTACHVGGGTATCTAAT (48), following the thermocycling steps: 94°C for 2 minutes, followed by 30 cycles of 98°C for 10 seconds, 62°C for 30 seconds, and 68°C for 30 seconds and a final extension at 68°C for 5 minutes. PCRs were performed in triplicate for each DNA extract. Amplicons were quantified using the ABI StepOnePlus Real-Time PCR System (Life Technologies, Foster City, USA). Purified amplicons were pooled in equimolar and paired-end sequenced (2 × 250 bp) using the Illumina HiSeq2500 platform. The resulting raw sequences were deposited at Mendeley Data (doi: 10.17632/f7vcfjt3j9.1).

### Sequence processing

The QIIME2 pipeline (version 2020.2) was used to analyze the 16S rRNA gene sequences (49). Raw data were filtered and denoised by the DADA2 plugin with default settings to generate amplicon sequence variants (ASVs) (50). ASVs, representing unique prokaryote taxa, were classified into taxonomic groups based on the SILVA v138 database using a feature classifier (https://www.arb-silva.de/documentation/release-1381/) (51). The ASV rarefaction curve was used to estimate the adequacy of the sampling depth (Fig. S1). ASVs annotated as chloroplasts, mitochondria, and archaea, as well as singletons, were removed from the data set. To ensure the follow-up analysis could carry on under the same standard, the dilution function of the Vegan package in R (52) was used to level the total number of sequences in each sample to the minimum number of sequences in all samples (53). One thousand bootstrap alignments of representative sequences were generated with the FFT-NS-i option in MAFFT (54). A phylogenetic tree was inferred using the FastTree algorithm (55) and converted to a rooted tree at the midpoint by MEGA 11 (56).

### Diversity, ecological processes, and statistical analyses

The *α*-diversity indices, including Chao1, Pielou, and Shannon, were calculated by the vegan package in R (52) to assess the degree of bacterial community diversity in each sample. One-way ANOVA and the least significant difference (LSD) test were conducted to detect significant differences in the α-diversity indices between different communities. Nonmetric multidimensional scaling (NMDS) analysis based on weighted Unifrac distance was performed to explore the clustering characteristics of microbial community composition between groups. Permutational multivariate analysis of variance (PERMANOVA) tests were used to examine statistically significant differences in the community structure and functional structure due to grouping (52). Differences in the β-diversity between microbial communities were compared using an unpaired *t*-test or Mann–Whitney U test. The ecological functions of each ASV in bacterial communities in the phycosphere were obtained using the functional annotation of prokaryotic taxa (FAPROTAX) (57). The clustering characteristics of microbial community function between groups were examined using NMDS based on the Bray–Curtis distance.

Bar plots were used to compare the proportion and variation tendency of dominant species in bacterial communities between groups at phyla and order levels. The STAMP program with Welch’s *t*-test was used to analyze statistically significant differences in taxa abundances between groups (58). Canonical correspondence analysis (CCA) was performed using the vegan package in R to gain deeper insights into the relationship between microbial community structure and environmental factors, including temperature, salinity, phosphate, nitrate and nitrite, as well as ammonium (59). Environmental parameters for the 2014 bloom were reported by Zhang et al. (45). In the 2018 bloom event, temperature, salinity, dissolved oxygen, and pH were determined using a multiparameter water quality meter (YSI6600V2). The nutrient concentrations (including phosphate, nitrate and nitrite, and ammonium) were determined using an autonomous nutrient analyzer (Bran-Lubbe AAIII, Germany) on seawater samples, which were filtered through 0.22 µm membranes and stored at −20°C until measurement in the laboratory. Variance partitioning analysis was performed to illustrate the contribution of environmental factors to CA and FL bacterial community variations (52).

The combination of the neutral community model (NCM) and null model was used to determine the assembly processes in the CA and FL communities (60). The NCM was employed to evaluate the potential impact of stochastic processes on the formation of CA and FL communities. The relative contributions of different mechanisms in shaping the CA and FL bacterial communities during the development of blooms were quantified using an inferred community assembly mechanism by phylogenetic-bin-based null model analysis (iCAMP) (61). In the iCAMP, the β-nearest taxon index (β-NTI) and Bray–Curtis-based Raup–Crick (RCbray) indices were used to quantify the contribution of deterministic [homogeneous selection (HoS), heterogeneous selection (HeS)] and stochastic [dispersal limitation (DL), homogenizing dispersal (HD), and drift (DR)] processes to bacterial community construction. Samples with β-NTI values < −1.96 or >1.96 were statistically significant, indicating that the relative effects of community turnover were determined by HoS or HeS, respectively. The samples with |β-NTI| < 1.96 were further analyzed by the RCbray index. The RCbray values of < −0.95, > +0.95, and between −0.95 and +0.95 signified HD, DL, and DR, respectively (61).

### Ecological network construction

Co-occurrence networks were constructed and visualized to investigate the interaction patterns of CA and FL bacterial communities under bloom conditions by using molecular ecological network analyses (MENA) (62) and Cytoscape (63). ASVs absent from 50% of the samples were removed to simplify the network. Node roles in networks were assessed using two key topological parameters: within-module connectivity (*Zi*) and among-module connectivity (*Pi*). *Zi* quantifies how well node *i* is interconnected with other nodes within its module, with higher *Zi* values indicating greater connectivity within that module. *Pi* assesses how “well-distributed” the links of node *i* are among different modules, with values close to 1 indicating a uniform distribution among all modules and values close to 0 reflecting a concentration of links within its module (64). ASVs were defined as network hubs when *Zi* > 2.5 and *Pi* >0.62, as module hubs when *Zi* > 2.5 and *Pi* ≤0.62, as connectors when *Zi* ≤ 2.5 and *Pi* >0.62, and as peripherals when *Zi* ≤ 2.5 and *Pi* <0.62 (65, 66). The first three are termed keystone species (66).

## RESULTS

### Environmental conditions and cell concentration

Both physical (temperatures and salinity) and chemical (phosphate, nitrate and nitrite, and ammonium) environmental parameters between the two bloom events were different in all aspects (Fig. 1B; Table S1). The Z station had lower temperatures and salinity but higher nutrient concentrations compared to the P station. The collected samples covered the pre-bloom and bloom stages. Microscopic examination and cell count results indicate that *P. shikokuense* was the dominant species in the blooms at both the Z and P stations (Table S2). There was a steady increase in the cell density of *P. shikokuense* from Z0 (0) to Z2 (8.9 × 10^6^ cells L^−1^) in the Z station samples, representing the bloom development process. The cell density decreased from P1 (1.9 × 10^7^ cells L^−1^) to P2 (8.2 × 10^6^ cells L^−1^), representing the bloom dissipation process (Fig. 1B). Other water sample characteristics, such as chlorophyll, dissolved oxygen, conductivity, pH, and average light intensity, were provided in Table S3.

### Diversity of bacterial communities

A total of 577,577 clean sequence reads were obtained across 30 samples of two bloom events after quality filtering (Table S4). The hierarchical clustering and taxonomic annotation indicated that samples Z2A-2 and P2A-3 were significantly different from the other two replicate samples from their respective sampling events and were excluded from comparative analysis (Fig. S2). A total of 10,157 ASVs retained after quality filtering from 28 samples were included in further analyses.

**Fig 2:**
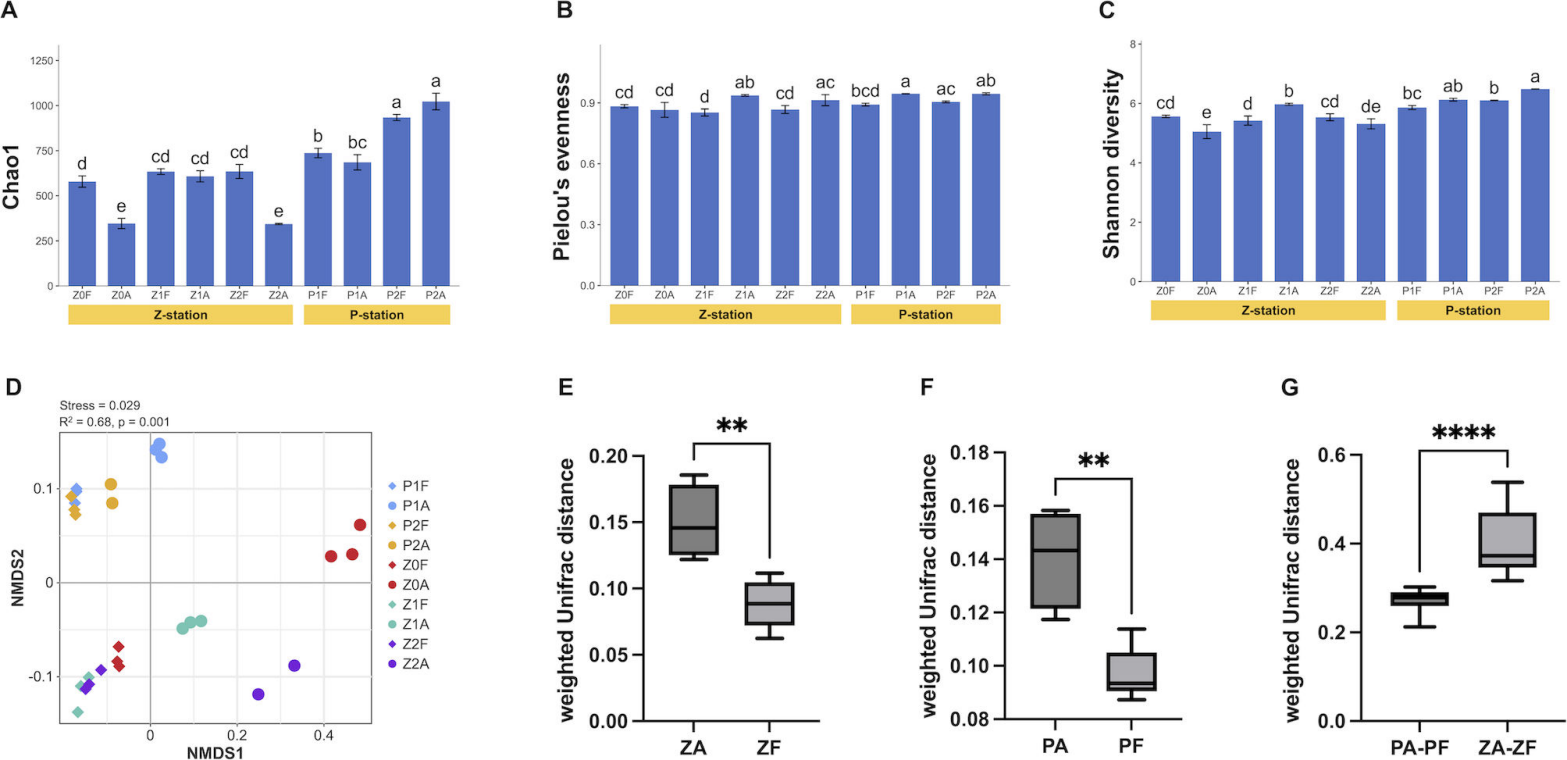
Differences in the alpha and beta diversity indices among different communities. (**A**) Chao1 index, (**B**) Pielou’s evenness, and (**C**) Shannon diversity of cell-associated (CA) and free-living (FL) communities from the two *P. shikokuense* bloom events. Error bars represent standard deviations. Comparison groups sharing the same letter indicate no significant differences, whereas groups without any shared letter indicate a significant difference following the LSD test (at a significance level of 0.05). (**D**) NMDS plot based on weighted Unifrac distance with PERMANOVA. Circles represent CA communities, diamonds represent FL communities, and different colors represent different groups. (**E, F**) Statistical analysis of weighted Unifrac distance between CA and FL communities; (**G**) the distance between PA-PF and ZA-ZF (**P < 0.01; ****P < 0.0001).

The α-diversity indexes showed different patterns between the CA and FL communities at different bloom stages (Fig. 2A through C). At the Z station, the Chao1 index of the CA community varied significantly as the bloom developed. The Chao1 index of the CA community was significantly higher during the early stages of the bloom (Z1) compared to the pre-bloom period (Z0), but it significantly decreased as the bloom progressed to the mid-bloom stage (Z2). In contrast, the FL community exhibited a stable Chao1 index, with no notable changes observed over the same period. At the P station, the Chao1 index for both bacterial communities increased significantly as the bloom collapsed, with no significant difference between the FL and CA communities. Pielou’s evenness index of the CA community was significantly higher than that of the FL community in the bloom stage (P1 and Z1). During the occurrence and development of both algal bloom events, Pielou’s evenness index and Shannon index of the FL community remained relatively stable, showing no significant differences across all groups. In contrast, the occurrence of algal bloom led to a significant increase in the Shannon index of the CA community (comparison between Z0A and Z1A).

NMDS analysis showed that each group is well clustered; particularly the FL communities gather together in accordance with different locations, but the CA communities exhibit relatively scattered distributions (PERMANOVA R^2^ = 0.68, *P* = 0.001) (Fig. 2D). The average weighted Unifrac distance among CA communities is significantly higher than that among FL communities in both bloom events (*P* < 0.01) (Fig. 2E and F), and the distance between CA and FL communities at the Z station was significantly greater than that at the P station (*P* < 0.0001) (Fig. 2G), whereas there is no significant difference between the pairwise comparison of either CA (ZA-PA) or FL (ZF-PF) communities, respectively (Fig. S3). The results showed that the phycosphere bacterial communities were similar between two spatially and temporally isolated *P. shikokuense* bloom events.

### Distinct composition between the FL and CA communities

**Fig 3:**
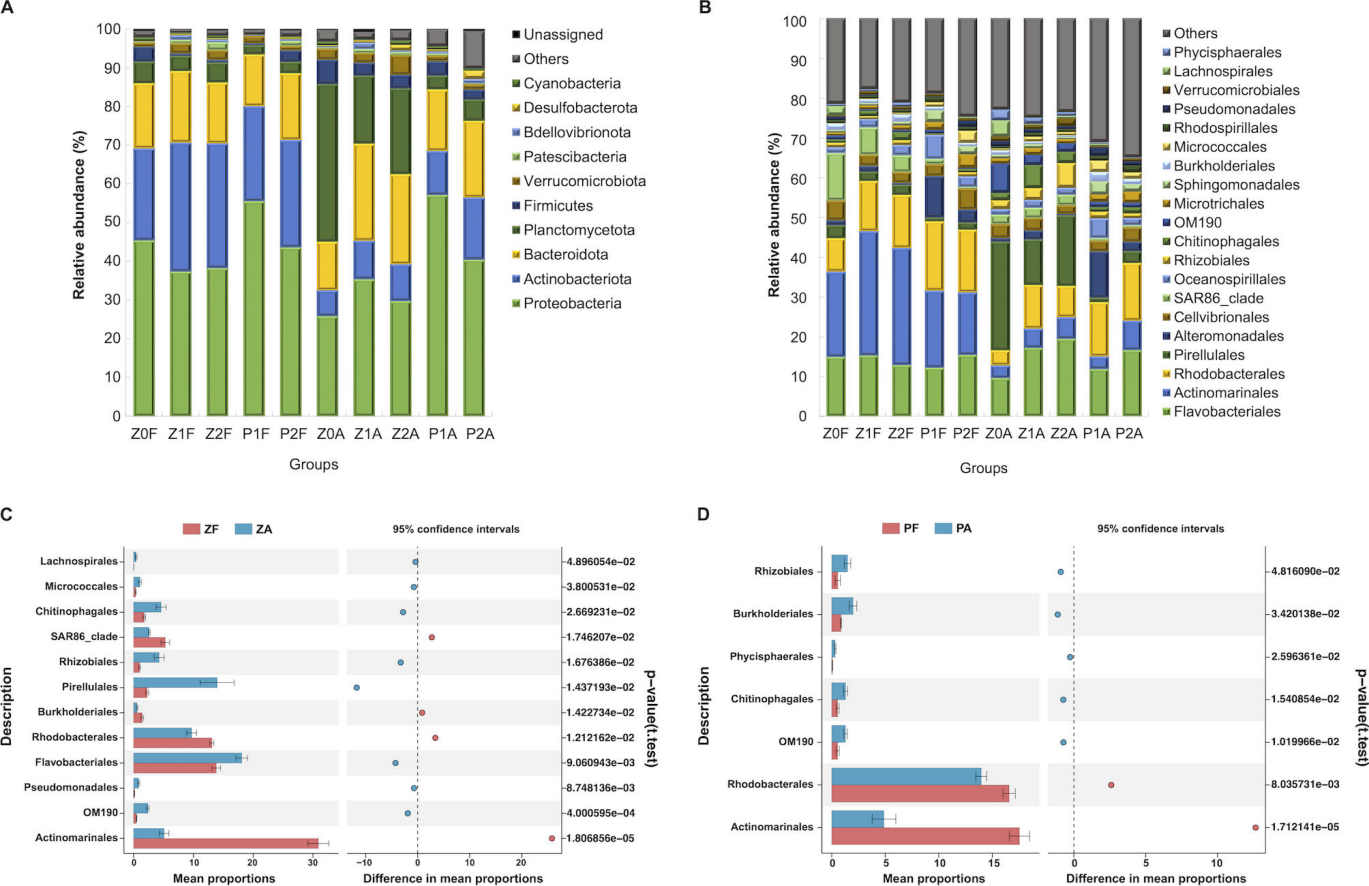
Relative abundance of taxa and their differences between CA and FL communities. Community composition of bacteria at (**A**) phylum and (**B**) order levels. The top 10 and top 20 taxa at phylum and order levels are shown. The others are combined and represented as “Others.” Top 20 orders with significant differences in the relative abundance between CA and FL communities from (**C**) PD14 and (**D**) PD18 events during the bloom (*P* < 0.05).

The top ten dominant phyla (89.78%–98.97% ASVs) were Proteobacteria, Actinobacteriota, Bacteroidota, Planctomycetota, Firmicutes, Verrucomicrobiota, Patescibacteria, Bdellovibrionota, Desulfobacterota, and Cyanobacteria in all groups (Fig. 3A). Overall, Proteobacteria had the highest relative abundance (29.74%–57.12%), followed by Actinobacteriota and Bacteroidota, together accounting for more than 80% ASVs in most samples. An exception was observed in the CA community at the Z station, where Planctomycetota was most abundant during the non-bloom phase (Z0) and maintained a high abundance during the bloom phase (17.57%–40.73%).

At the order level, the CA and FL bacterial compositions differed markedly in both events (Fig. 3B). In the FL communities of both events, Actinomarinales and Rhodobacterales comprised the majority, with significantly higher abundance than those in the CA communities (Fig. 3C and D), whereas there were different patterns in the CA communities. In the Z station samples, the proportion of Pirellulales is the highest (27.27%) in the Z0A, which then decreased to an average of 14.61% in the Z1A and Z2A (Fig. 3B). In the P station samples, Pirellulales only accounted for an average of 2.09% in the P1A and P2A. Instead of that, Alteromonadales accounted for 11.57% in the P1A, which then decreased to 2.38% in the P2A.

**Fig 4:**
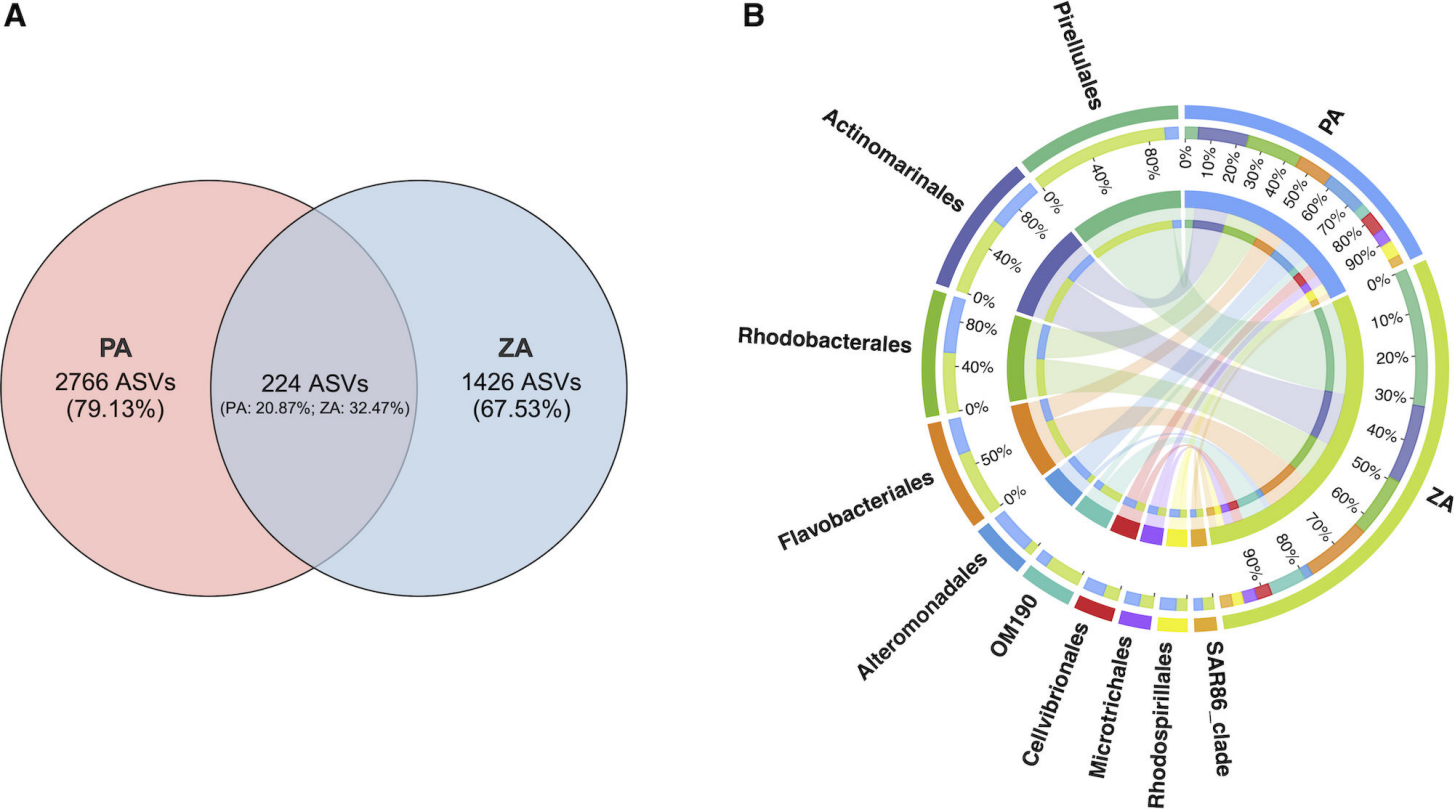
The CA bacteria common to the two algal bloom events. (**A**) Venn diagram displays the number and relative abundance of ASVs as commonly existing in ZA and PA communities. The percentages in parentheses indicate the relative abundance of unique or shared ASVs within their respective groups. (**B**) The Circos diagram displays the co-taxa (top 10) between ZA and PA communities at the order level. The left half-circle represents bacterial orders, with the color of the circle marked with scales indicating different groups and the length of the arc representing the distribution ratio of the groups within a certain taxon. The right half-circle represents groups, with the color of the circle marked with scales indicating different taxa and the length of the arc representing the distribution proportion of taxa within the corresponding group. The innermost lines in the middle indicate the connections between the taxa and groups, with thicker lines representing a higher abundance of the corresponding taxa.

The Venn diagram showed that the CA communities of two events shared 224 ASVs, accounting for 20.87% relative abundance at the P station and 32.47% at the Z station (Fig. 4A). These ASVs were primarily distributed among the orders Rhodobacterales, Flavobacteriales, and Alteromonadales. In addition, a large number of ASVs were affiliated with Pirellulales, Actinomarinales, OM190, Cellvibrionales, Microtrichales, and Rhodospirillales (Fig. 4B).

Comparison between the samples of non-bloom and bloom stages showed that the occurrence of blooms significantly increased the abundance of Chitinophagales and Rhodobacterales and decreased the abundance of Lachnospirales in both CA and FL communities (Fig. S4).

### Effects of environmental factors and ecological processes shaping the bacterial community

**Fig 5:**
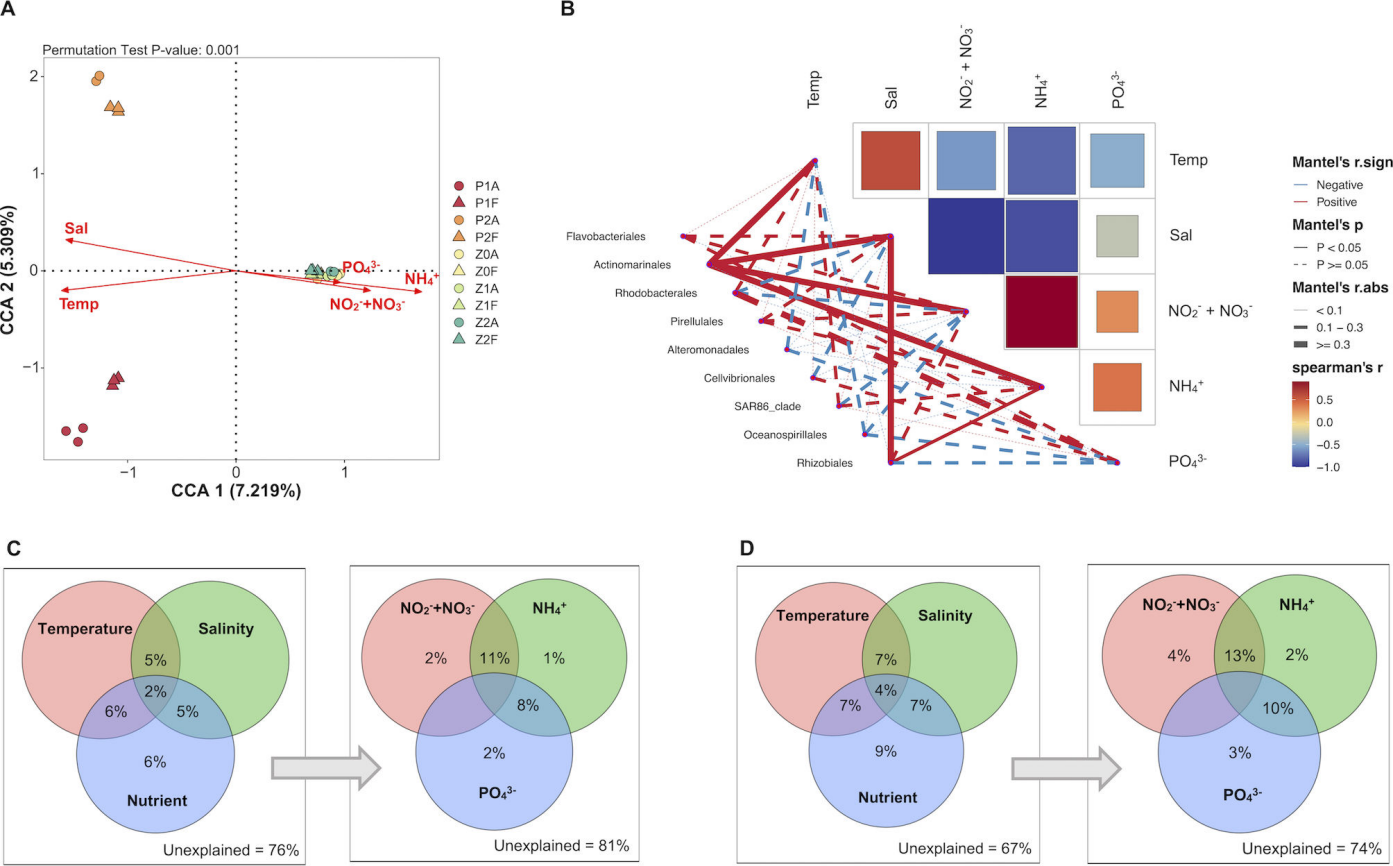
The impact of environmental factors on bacterial communities. (**A**) The relationship between environmental factors and bacterioplankton community was evaluated by CCA. (**B**) The relationship between bacterial abundance and environmental factors at the order level was analyzed using the Mantel test. Line widths represent the absolute values of the correlation coefficients (**R**), while colors indicate the direction of the correlation (positive or negative). Solid and dashed lines are used to indicate statistical significance. Relative influences of environmental variables on the (**C**) CA and (**D**) FL communities based on VPA (contributions ≤ 0 are not displayed).

Environmental factors play an important role in shaping bacterial communities in terms of the deterministic process. CCA showed that inorganic phosphate and nitrogen (nitrate, nitrite, and ammonium) are strong drivers contributing to the variation in bacterial communities at the Z station (Fig. 5A; Table S5). Mantel analysis showed that Actinomarinales exhibited a significant positive correlation with temperature, salinity, and inorganic nitrogen, and Rhizobiales showed a significant positive correlation with salinity and ammonium (Fig. 5B; Table S6). Furthermore, Mantel analysis revealed significant positive correlations between the environmental factors and common ASVs of the CA communities shared by two independent bloom events. Besides the positive correlation with temperature, salinity, and inorganic nitrogen as observed in Pirellulales and OM190, Flavobacteriales were also positively correlated with phosphate (Fig. S5; Table S7).

Variation partitioning analysis (VPA) revealed that environmental factors could explain a relatively small proportion of the variations in the community structure, and FL communities (33%) were more affected by environmental factors than CA communities (24%) (Fig. 5C and D). Among those, nutrients accounted for 9% and 6% of the variation in community composition in the FL and CA communities, respectively. Physical factors (temperatures and salinity) contributed weakly to both CA and FL community variations.

**Fig 6:**
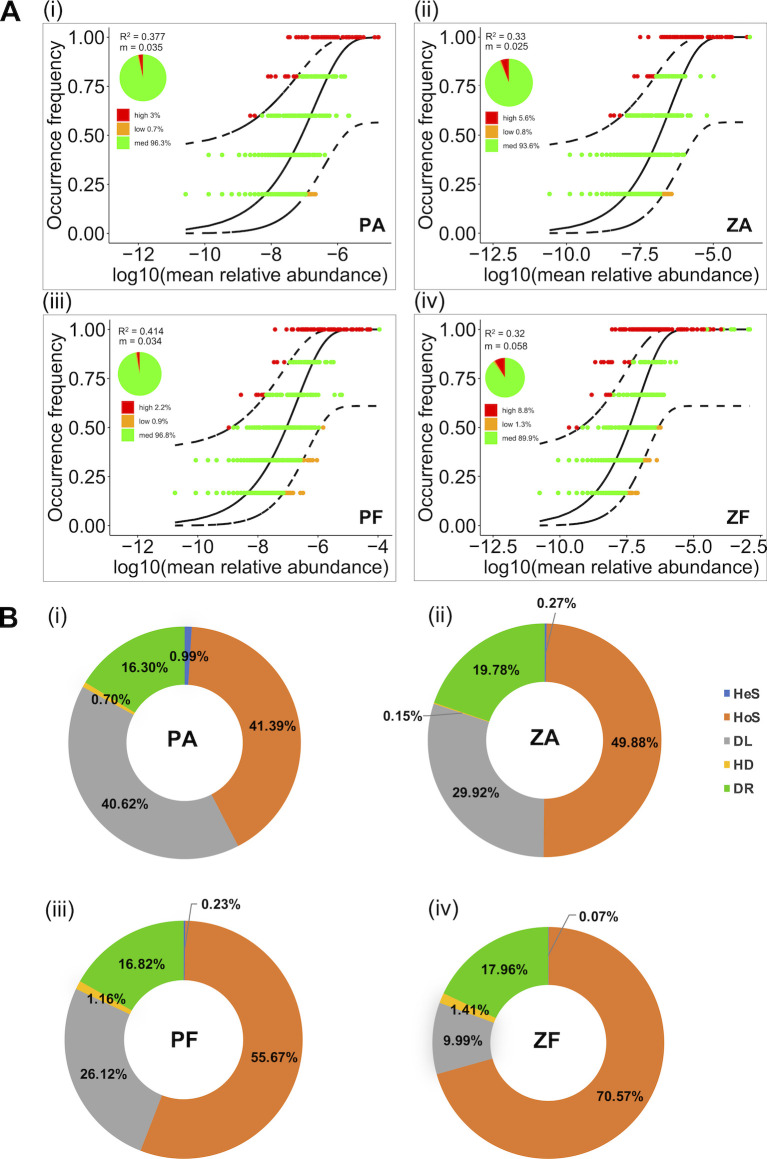
The relative impacts of ecological processes on community assembly. (**A**) Fit of the neutral models for the microbial communities, red dots represent observed ASV frequencies higher than model predictions (high), orange dots represent observed ASV frequencies lower than model predictions (low), black dotted lines represent 95% confidence intervals around model predictions, and green dots represent ASVs within confidence intervals (med). (**B**) The relative importance of ecological processes to the construction of bacteria communities. Values in the figure are derived by averaging the importance of ecological processes, which are determined based on null model analysis for each sample. HeS: heterogeneous selection; HoS: homogeneous selection; DL: dispersal limitation; HD: homogenizing dispersal; DR: drift.

The Sloan neutral model (NCM) was used to appraise the contribution of stochastic processes in community assembly (Fig. 6A). Higher fitting values (R^2^) were observed in both communities at the P station (0.377; 0.414) than at the Z station (0.32; 0.33), indicating a greater influence of stochastic processes. Meanwhile, the migration rate was lower in the ZA (0.025) community than in the ZF community (0.058), suggesting higher dispersal limitation in CA compared to that in FL at the Z station.

Furthermore, a null model-based iCAMP analysis was used to quantify the contributions of ecological processes to the community assembly (Fig. 6B; Table S8). All the detected 10,157 ASVs were clustered into 372 phylogenetic bins by iCAMP. The top taxa in each major bin were *Candidatus_Actinomarina*, *Blastopirellula*, Rhodobacteraceae, *SAR86_clade*, *Planktomarina,* and Flavobacteriaceae (Fig. S6). As described in the model principles, HeS and HoS are considered to be deterministic, while DL, DR, and HD are regarded as stochastic processes (67). Our results showed that, among the five components, HoS, DL, and DR played the dominant roles in driving the community assembly (Fig. 6B). Overall, the contribution of the deterministic process HoS was lower in the CA community (41.39%; 49.88%) than in the FL communities (55.67%; 70.57%). All eight top bins were mainly shaped by HoS, showing no effect of DL (Table S9). Consistent with the NCM analysis, a much higher ratio of the stochastic process (DL mainly) was observed in both PA (40.62%) and PF (26.12%) than those at the Z station (29.92% in ZA; 9.99% in ZF). Specifically, there were more bins dominated by DL in the FL communities at the P station (107–124 bins) than at the Z station (51–68 bins) (Table S10). Notably, the effect of DL was more significant in the CA communities compared with the FL communities in both events.

### Comparison of co-occurrence networks and prediction of ecological functions

**Fig 7:**
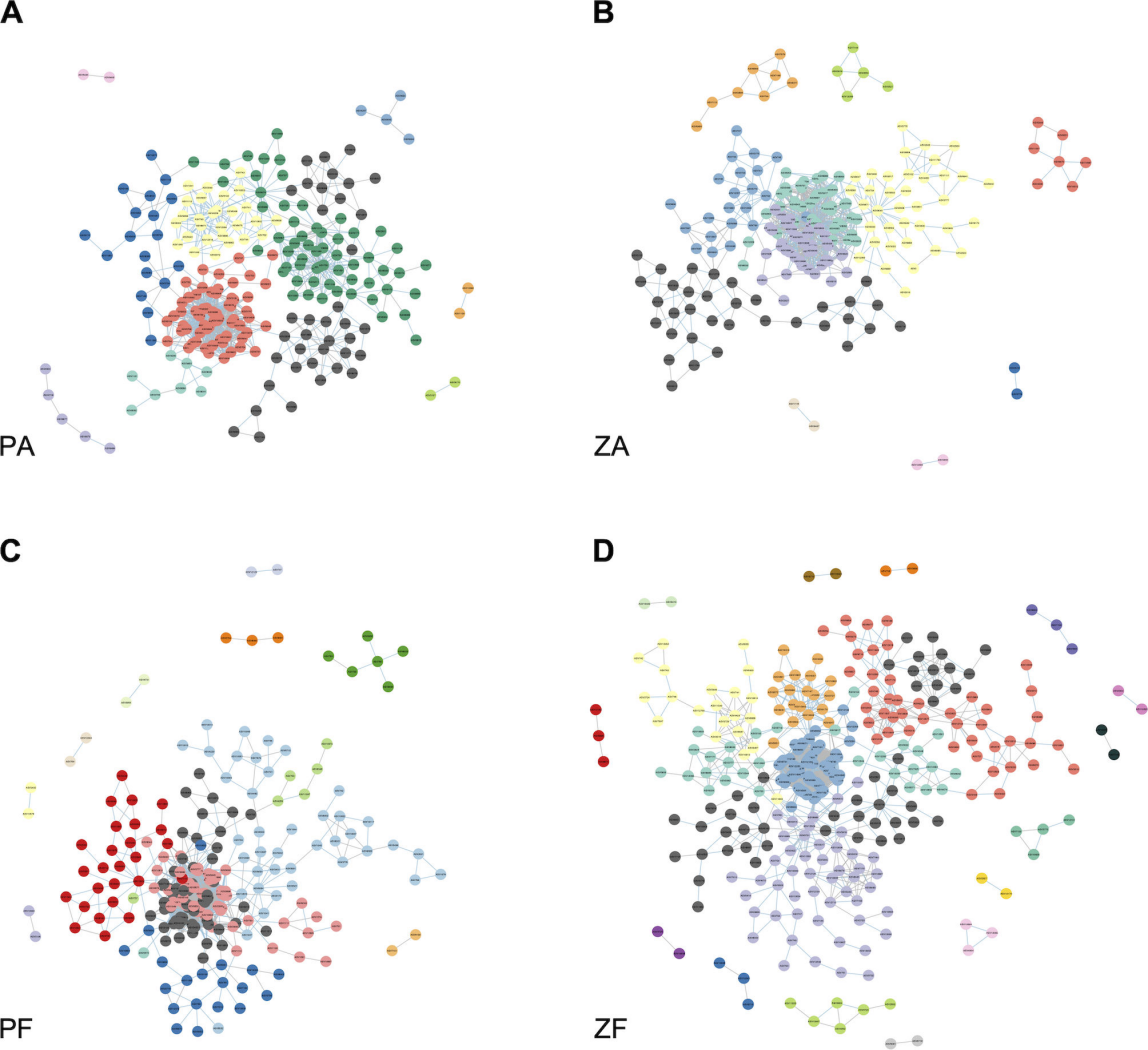
Co-occurrence patterns of the (**A**) PA; (**B**) ZA; (**C**) PF; and (**D**) ZF communities. Nodes represent ASVs, distinguished by colors based on modules. The blue and gray lines indicate positive and negative correlations, respectively.

Co-occurrence network analysis of bloom samples revealed that lower numbers of nodes, edges, and modules were identified in networks of both CA communities compared to those of FL communities, suggesting a simpler microbial interaction among the CA community (Fig. 7; Table S11). Five ASVs were identified as the module hubs in the PA network, including Terasakiellaceae sp., *Candidatus_Actinomarina*, *Formosa*, *Sva0996_marine_group,* and *Erythrobacter*. Three ASVs were identified as the module hubs in the ZA network, namely, *Maribacter* and *NS4_marine_group* from Flavobacteriaceae and one species from Rhodobacteraceae. Three ASVs were identified as keystone species in the PF network, namely, two connectors *OM60 (NOR5)_clade* and *PeM15* and one module hub *OM60 (NOR5)_clade*. Four ASVs were identified as keystone species in the ZF network, namely, three connectors *NS5_marine_group*, *Amylibacter,* and *Aurantivirga* and one module hub *NS4_marine_group* (Fig. S7).

**Fig 8:**
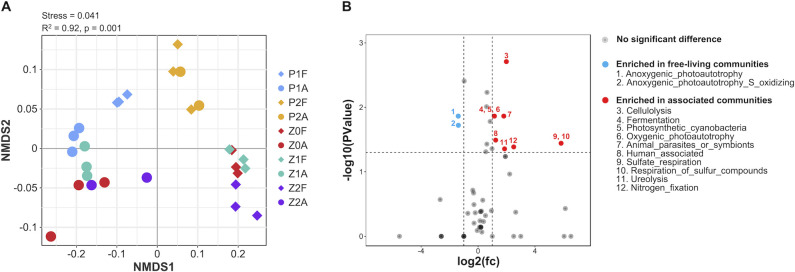
Ecological function characterization of CA bacteria compared with FL bacteria. (**A**) NMDS analysis of predicted ecological functions with PERMANOVA. (**B**) Enrichment of functions in CA (red) and FL (blue) communities is shown by the volcano plot. Red and blue dots indicate the functions in the CA community that are at least twofold higher and lower than detected in the FL community, respectively, with a *P*-value < 0.05 in the rank-sum test.

**Fig 9:**
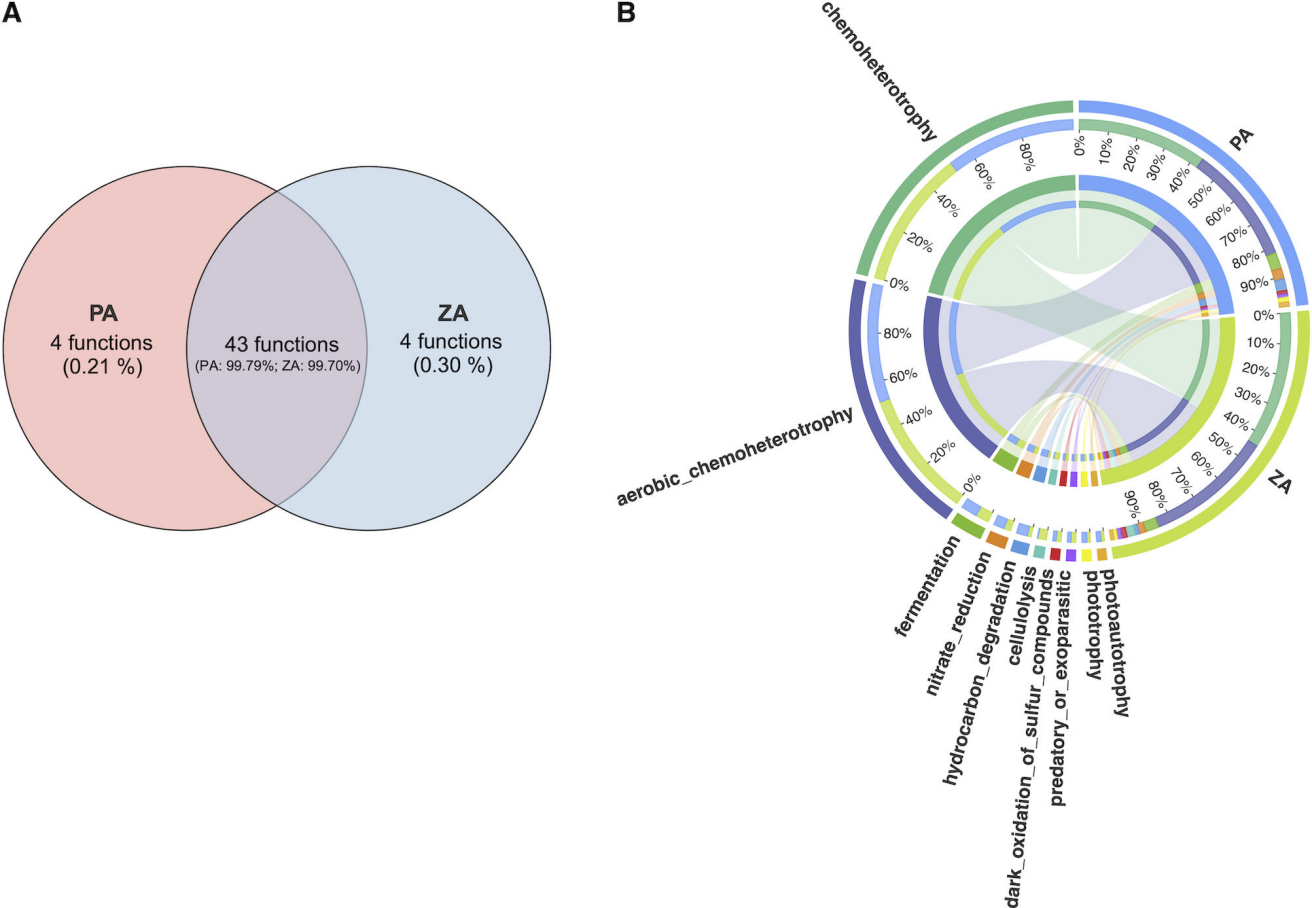
The predicted functions of CA bacteria common to the two algal bloom events. (**A**) The Venn diagram displays the number and relative abundance of functions shared between the ZA and PA communities. The percentage in parentheses indicates the relative abundance of unique or shared functions within each group. (**B**) The Circos diagram shows the top 10 functions shared between the ZA and PA communities. The left side represents the functions, with the circle marked with scales indicating the groups and the length of each arc corresponding to the distribution ratio of the group within a particular function. The right side represents groups, with the circle marked with scales indicating the functions and the length of each arc corresponding to the distribution proportion of each function within the respective group. The innermost lines in the center indicate the connections between the functions and groups, with a thicker line representing a higher abundance of the corresponding function.

Predicted metabolic functions of all ASVs cover the elemental metabolism of carbon, nitrogen, and sulfur, 59 in total, and each group is well clustered based on the NMDS analysis (PERMANOVA R^2^ = 0.92, *P* = 0.001) (Fig. 8A; Table S12). Notably, the function-based NMDS distance of the CA communities in two independent bloom samples was more closely clustered than the ASV-based NMDS distance (Fig. 2D), suggesting that the CA communities share similar functions despite different community compositions. Significant function differences between the CA and FL communities during the bloom phase were identified as well (Fig. 8B). Functions related to nutrient and energy metabolism, cellulolysis, and symbionts were significantly enriched in the CA community. The Venn diagram revealed that CA communities of both events share 43 common functions, which account for the majority of each event (Fig. 9A). These shared functions are primarily associated with chemoheterotrophy and aerobic_chemoheterotrophy. Additionally, a significant number of functions related to fermentation, nitrate_reduction, hydrocarbon_degradation, cellulolysis, dark_oxidation_of_sulfur_compounds, predatory_or_exoparasitic, as well as phototrophy and photoautotrophy, were also present (Fig. 9B).

## DISCUSSION

Two independent and spatially distanced *P. shikokuense* bloom events served as good duplicates to study the dynamic shift of the bacterial community and assembly mechanism in the natural environment, especially for those in the phycosphere. Our results showed different bacterial community structures between bloom and non-bloom. Significant differences in the composition, structure, function, and assembly mechanisms between the CA and FL bacterial communities were identified.

### Composition characteristics of the bacterial communities in two algal bloom events

Beta diversity analysis revealed that the differences in the weighted Unifrac distance between FL and CA communities were greater than between sampling sites and bloom status, together with the higher contribution of DL observed in CA communities, indicating that the phycosphere reshaped the microbial communities.

The algal phycosphere is the region enriched with organic exudates that attract and stimulate microbial communities. The phycosphere radius is influenced by factors such as phytoplankton cell size, exudation rates, growth rates, and turbulent environment (12), with the radius of *Prorocentrum shikokuense* estimated to range from 10 to 10^3^ µm based on its cell size. Bacterial communities are sensitive to environmental changes (68). Our results indicate that the bacterial community associated with the *P. shikokuense* cells underwent dynamic changes during the development of algal blooms, showing a distinct pattern compared with the FL bacterial community in the surrounding waters. In the non-bloom phase (Z0 sample), the phytoplankton community was dominated by diatoms (45) and Planctomycetota was found to be the most abundant phyla in the CA community correspondingly in this study. As suggested, there may be a mutualism between the Planctomycetota and diatom, which promotes the occurrence of a diatom bloom (69). Consistent with that, Pirellulales and OM190 (both from Planctomycetota) were enriched in the diatom-dominated stage, and the relative abundance decreased sharply with the occurrence of *P. shikokuense* bloom at the Z station. The occurrence of blooms of *P. shikokuense* at both sites corresponded with a significant increase in the relative abundance of Chitinophagales and Rhodobacterales. This is consistent with the report that they can degrade complex organic substances, which leads to their prolific growth during blooms due to the increase in phytoplankton-derived metabolites (70–72).

A great abundance of Rhizobiales was detected in the CA communities during the development of bloom at the Z station and the P station in this study. Rhizobiales can fix nitrogen (N_2_) and convert it into ammonia and other organic nitrogen compounds that could be utilized by plants. In recent years, studies have found associations between Rhizobiales and marine algae, such as green algae (73, 74), brown algae (75, 76), cyanobacteria (77), and dinoflagellates (78). A *Rhizobium* bacterium was isolated from the dinoflagellate *Gambierdiscus balechii*, and the abundance increased when the algae grew under nitrogen limitation conditions (79). A recent study found that the Rhizobiales bacterium Candidatus *Tectiglobus diatomicola,* capable of nitrogen fixation, formed a symbiotic relationship with diatoms (80). It provides fixed N to the diatom host and acquires carbon sources produced through the host’s photosynthesis. *Rhizobium* sp. has also been confirmed to exhibit positive hydrolysis activity for various types of carbon, nitrogen, and phosphorus (81). Importantly, correlation analysis indicated that Rhizobiales was significantly positively correlated with ammonium (Fig. 5B), reinforcing the argument that the presence of such bacteria may provide sufficient nutrients for the growth of algae and thus promote algal blooms, although the species associated with dinoflagellates have not been shown to fix N_2_ (79).

In addition, a high relative abundance of Alteromonadales and Oceanospirillales was detected, with a noted decrease in their relative abundance during the dissipation stage of algal blooms (P1 to P2). According to previous studies (82–84), many bacterial species in these two orders can inhibit the activity or kill the algal cells by releasing metabolites with algicidal activity or by digesting the cell wall polysaccharides to lyse the algal cells. In particular, *Alteromonas macleodii* (strain FDHY-03) was found to exhibit algicidal activity against *P. shikokuense* by digesting its cell wall polysaccharides (85). As a result, they might function in the demise process of the bloom.

### Environmental conditions, interspecific interactions, and assembly mechanism

Niche-based theories assume that environmental conditions (e.g., pH, temperature, and salinity) and interspecies interactions (e.g., competition, predation, and reciprocity) control community structure, often referred to as deterministic processes (26, 27). The analysis of VPA indicated that environmental factors drive the FL community assembly more strongly than the CA community during the bloom (Fig. 5C and D). This is in line with the lifestyle of the FL bacteria, which drifts in the waters and is easily influenced by environmental factors. Consistent with previous studies (29, 86), only partly (19%–33%) bacterial community variation was explained by environmental parameters, indicating that other undetermined factors are important contributors to the variation of community structure. Co-occurrence patterns showed a more complex and stable network in the FL communities than the CA communities, suggesting more interspecific interactions occur in FL communities. Compared with the FL community, the CA community was less affected by environmental changes and had fewer co-occurrence and co-exclusion relationships.

The NCM and null model analysis revealed that the effects of deterministic processes (mainly HoS) in FL communities were greater than in CA communities during *P. shikokuense* bloom. This is consistent with the reports of previous studies on the mechanism of particle-attached and free-living microbial community assembly in the marine environment (87, 88). It was found that HoS, DL, and DR together dominate the microbial community assembly according to the iCAMP model. The relative importance of HoS was higher in the FL than in the CA communities, which was attributed to the different habitats. During an algal bloom, algal cells are coated with a layer of secretions (89), while the surrounding seawater is more homogeneous and hydrologically connected. This may also explain the higher ratio of HoS at the Z station than at the P station, given that the Z station is located in the Changjiang estuary featuring more active hydrodynamic conditions. As a result, FL bacteria could move and disperse more freely in surrounding water. In contrast, the CA communities are subjected to greater dispersal limitations, due to the physical attachment to the algal cells or the physiological selection dependent on the interaction in the phycosphere. Thus, the higher contribution of DL at the P station than that of the Z station may be caused by the release of a large amount of organic matter and nutrients by algal cells during the bloom dissipation.

### Shared metabolic function in the CA communities and ecological implications

The study found that the CA bacterial communities were less similar at the taxonomic level than the predicted functional composition, in line with reported findings in the study of bacterial communities associated with the green macroalga *Ulva australis* (90). The competitive lottery model is applicable to address this phenomenon, in which more than one species can play a specific role in the ecosystem. Thus, bacteria with similar functions can occupy the same ecological niche (91, 92). It has been documented that different size-fractionated bacteria communities possess different functional patterns (93). Likewise, in this study, functional groups capable of S oxidation and anoxygenic photoautotrophy were significantly enriched in the FL community compared to the CA community. The growth of anoxygenic photoautotrophic bacteria generally relies on simple inorganic compounds (94), which are usually derived from the degradation of high-molecular weight compounds by the large-sized microbiota and gradually transition to the small-sized microbiota (20). Thus, these substrates are more accessible to FL bacteria due to their free-living nature, which allows for greater mobility. In contrast, CA bacteria are physically attached to algal cells and have more restricted access to available substrates.

Different from that, the abundance of the nitrogen fixation function and ureolysis were significantly higher in the CA bacterial community. The rapid growth of phytoplankton is dependent on the nitrogen supply. Microbial nitrogen fixation can help overcome N limitation, as discussed above (95). In addition, urea can be used as an extra nitrogen source to support the growth by *P. shikokuense* (96). Therefore, the existence of the CA bacteria involved in the nitrogen cycle may play a certain role in the outburst and maintenance of the *P. shikokuense* bloom.

Significant enrichment of the functional group related to cellulolysis was also identified in the CA bacterial community. Extracellular enzymes (such as xylanase, mannase, and cellulase) secreted by bacteria can decompose polysaccharides in the algae cell walls (97, 98). Many algicidal bacteria are able to adhere to algae cells and subsequently degrade large molecules on the cell surface (99). Bacteria with algicidal capability were more abundant in the late stage of bloom. The enrichment of cellulolysis in the CA community and the prevalence of algicidal bacteria in late bloom suggest that some algae-associated bacteria may be involved in the demise process of the algal bloom.

During both bloom events, among the common predicted functions of CA communities, the dominance of chemoheterotrophy, aerobic_chemoheterotrophy, and fermentation aligns well with the ecological context of massive algal-derived organic matter release during algal growth and death processes. Shared ASVs were primarily concentrated in the orders Rhodobacterales and Flavobacteriales (Fig. 4B), which have been found to be correlated with phytoplankton blooms (100, 101). Both of these orders have a high demand for organic nutrients and efficient degradation of complex organic compounds, enabling them to rapidly accumulate in nutrient hotspots (101–104). Therefore, these taxa may be key contributors to the cycling of organic material released by algae through chemoheterotrophy and fermentation. In addition, the function of nitrate_reduction was shared by both events, and Flavobacteriales, OM190, and Pirellulales shared by the two events showed a significant positive correlation with nitrogen nutrients (Fig. S5). As documented, certain species of Flavobacteriales and Pirellulales are capable of nitrate reduction (105–108). This suggests that these orders may play a critical role in the nitrogen cycling during the bloom, potentially through the nitrate_reduction process. In summary, common ASVs and ecological functions shared by the two independent bloom events underscore the central role of these microbial taxa in organic matter decomposition and nutrient cycling, which in turn can influence the processes of algal blooms. Thus, in further research, metagenomic and metabolomic approaches should be applied to elucidate the interaction mechanism between key bacteria taxa and the algal host.

### Conclusions

In this study, comparative analyses were conducted, including the community compositions, assembly mechanisms, and ecological functions between the CA and FL bacterial communities of two *P. shikokuense* bloom events. The results indicated that the compositions and structures of CA bacterial communities were significantly different from the corresponding FL communities in the surrounding seawater. Actinomarinales dominated FL communities, while the abundance of Actinomarinales in corresponding CA communities was reduced considerably. Further analysis showed that Pirellulales, Actinomarinales, and Rhodobacterales represent the main taxa in the CA communities shared by two bloom events and an increase of Rhizobiales as well. The effect of environmental factors (deterministic process) can explain a relatively small fraction of the variation in the community structure, higher in FL than CA. FL communities were mainly driven by homogeneous selection. The effect of dispersal limitation (stochastic process) on the CA community was greater than that on the FL community. This can be well explained by the phycosphere characteristics, where the physical attachment of CA bacteria to algal cells and physiological selection through interactions in the phycosphere both contribute to creating stronger dispersal limitations for CA communities. Different from the FL communities, the metabolic functions related to nutrient cycling and cell lysis were significantly enriched in the CA bacterial community, indicating that the phycosphere bacteria may play an essential role in the development and dissipation of algal bloom events. The insights gleaned from these two independent bloom events have enriched our understanding and provided novel insights into the dynamical shift of bacterial communities and the algae-bacteria interaction.

## Data Availability

The original data from 16S rRNA gene amplicon sequencing of this study are available at Mendeley Data (doi: 10.17632/f7vcfjt3j9.1).

## SUPPLEMENTAL MATERIAL

Supplemental figures spectrum.02451-24-s0001.pdf Fig. S1 to S7.

Supplemental tables spectrum.02451-24-s0002.xlsx Tables S1 to S12.

ASM does not own the copyrights to Supplemental Material that may be linked to, or accessed through, an article. The authors have granted ASM a non-exclusive, world-wide license to publish the Supplemental Material files. Please contact the corresponding author directly for reuse.
