# Supplementary material for: Unraveling the assembly mechanisms and differentiated ecological functions of protist cell-associated and free-living bacterial communities during two Prorocentrum shikokuense blooms
Source: Microbiol Spectr. 2025 May 15;13(6):e02451-24. doi: 10.1128/spectrum.02451-24 (PMC12131754; doi:10.1128/spectrum.02451-24)
Supplement: Supplemental figures — Fig. S1 to S7. [file spectrum.02451-24-s0001.pdf]

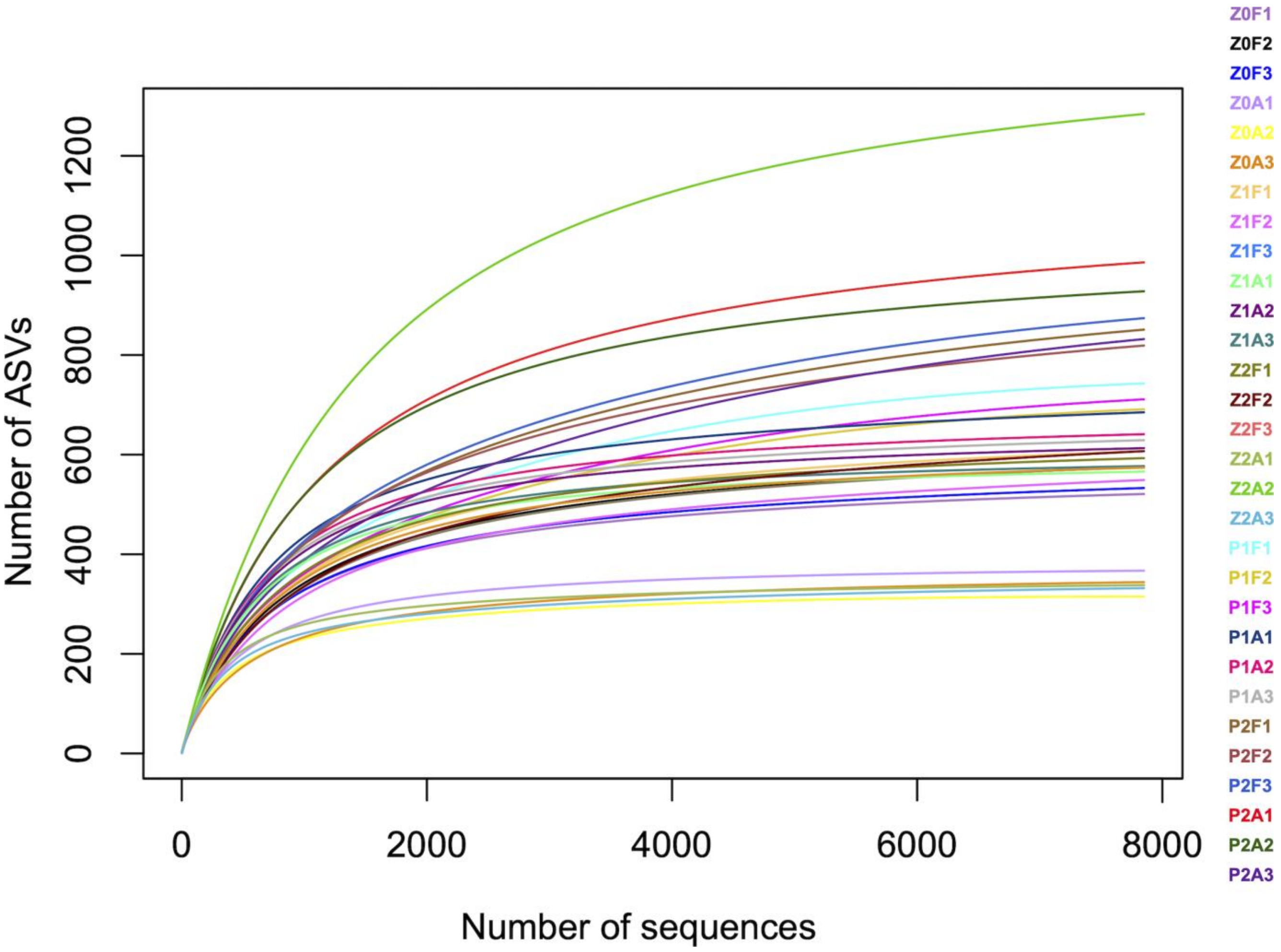

Fig. S1 **Rarefaction curve of detected ASVs of all the samples.**

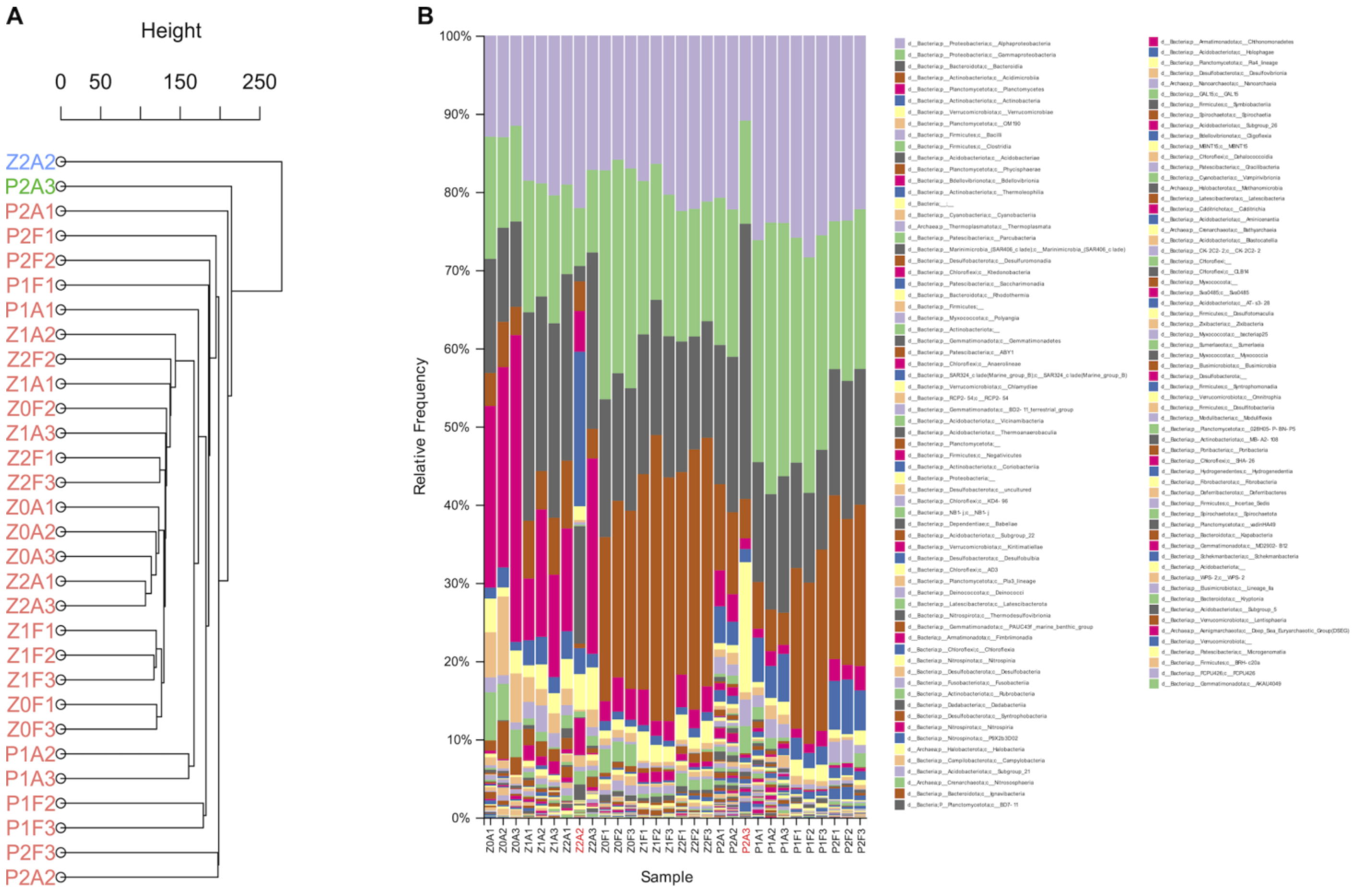

Fig. S2 **Hierarchical clustering and species annotation at the order level of all 30 samples.**

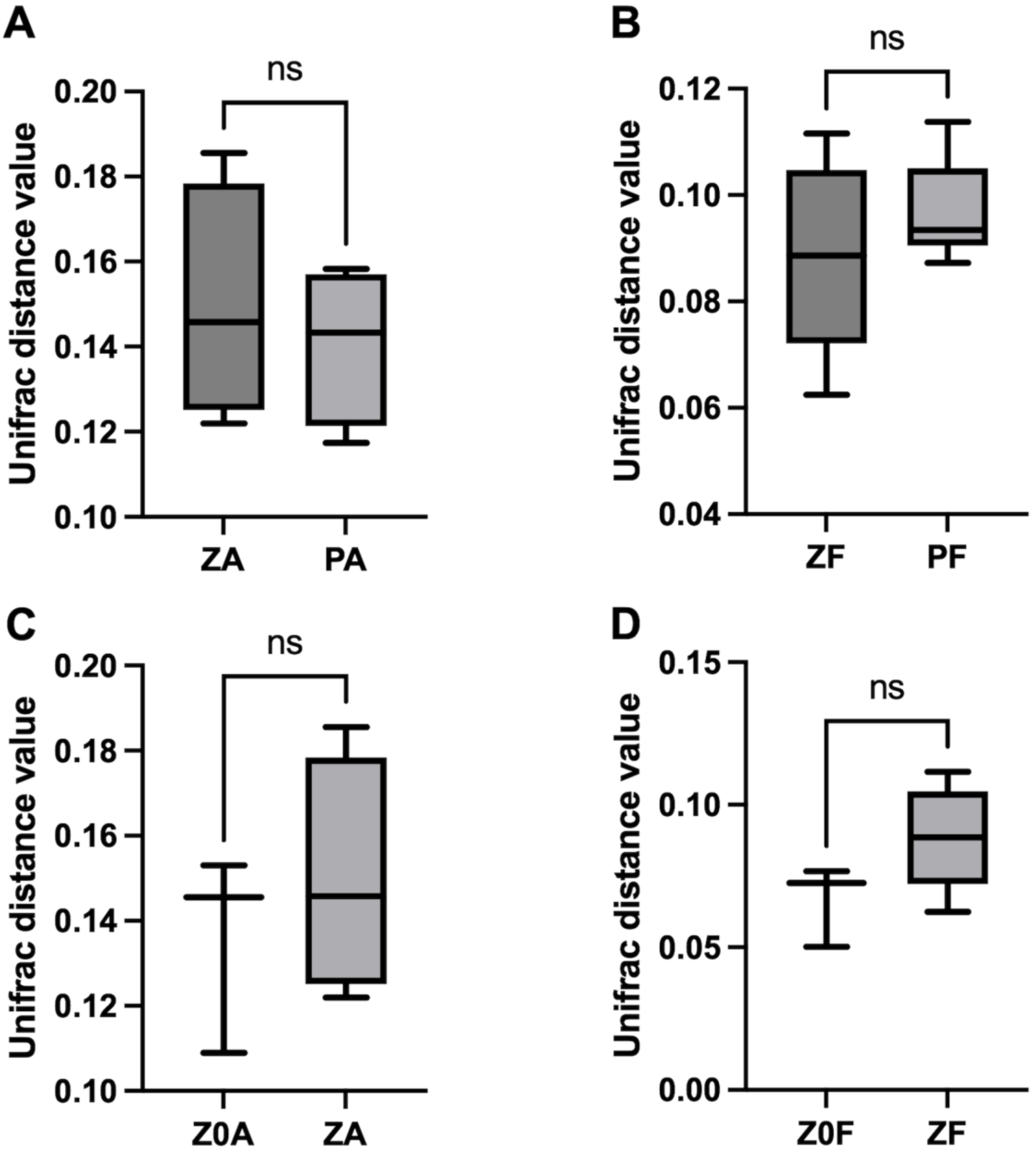

Fig. S3 **Statistical difference analysis of weighted Unifrac distance between Z and P stations during the blooms in (A) CA and (B) FL communities, and between non-bloom and bloom stages at Z station in (C) CA and (D) FL communities.**

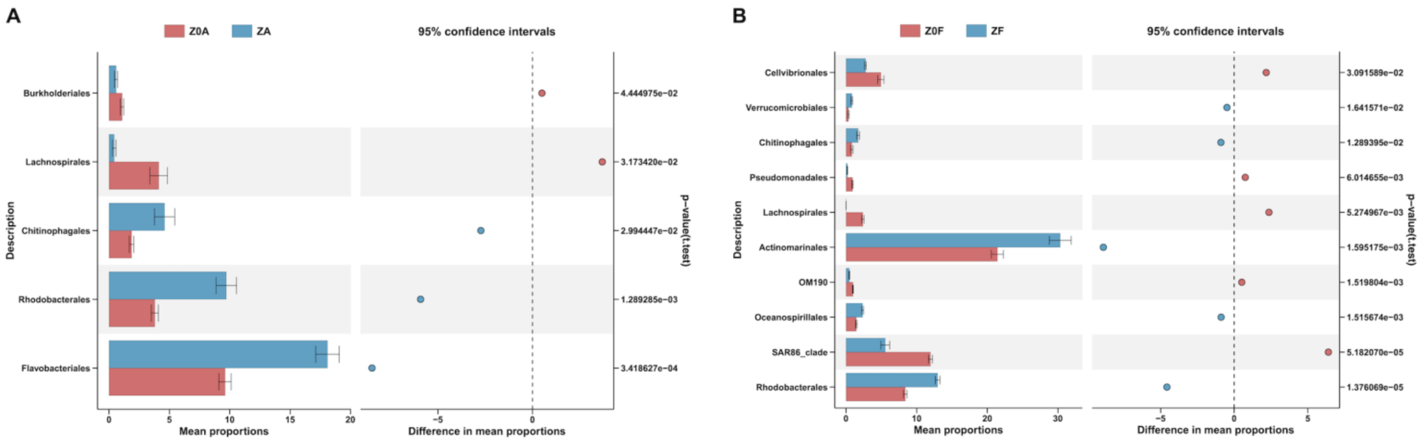

Fig. S4 **Top 20 orders with significant differences in relative abundance between non-bloom and bloom stages in (A) CA and (B) FL communities.** ($p < 0.05$)

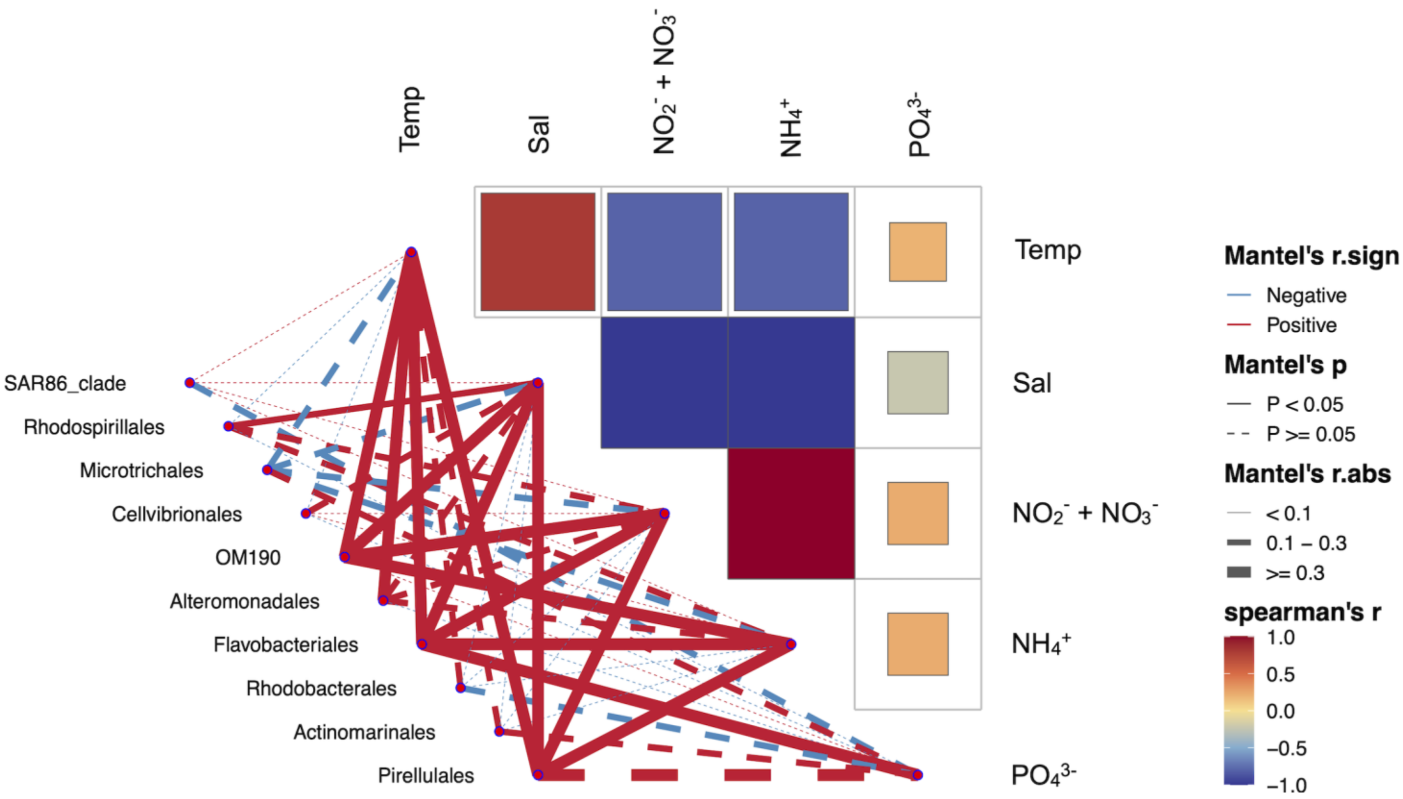

Fig. S5 **Correlation analysis using Mantel test between environmental factors and common ASVs shared by CA communities from two bloom events.** Red lines represent positive correlations, and blue lines indicate negative correlations. Line thickness reflects the absolute value of the correlation coefficient (R), with solid lines representing significant correlations and dashed lines indicating non-significant correlations.

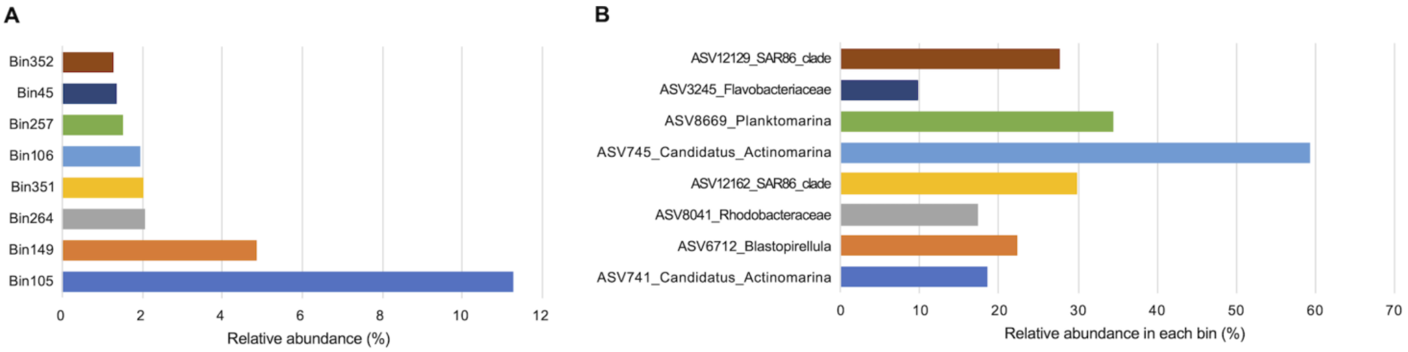

Fig. S6 **Relative abundance of (A) the top bins and (B) the top ASVs in each bin.** The same bin is shown in the same color in (A) and (B).

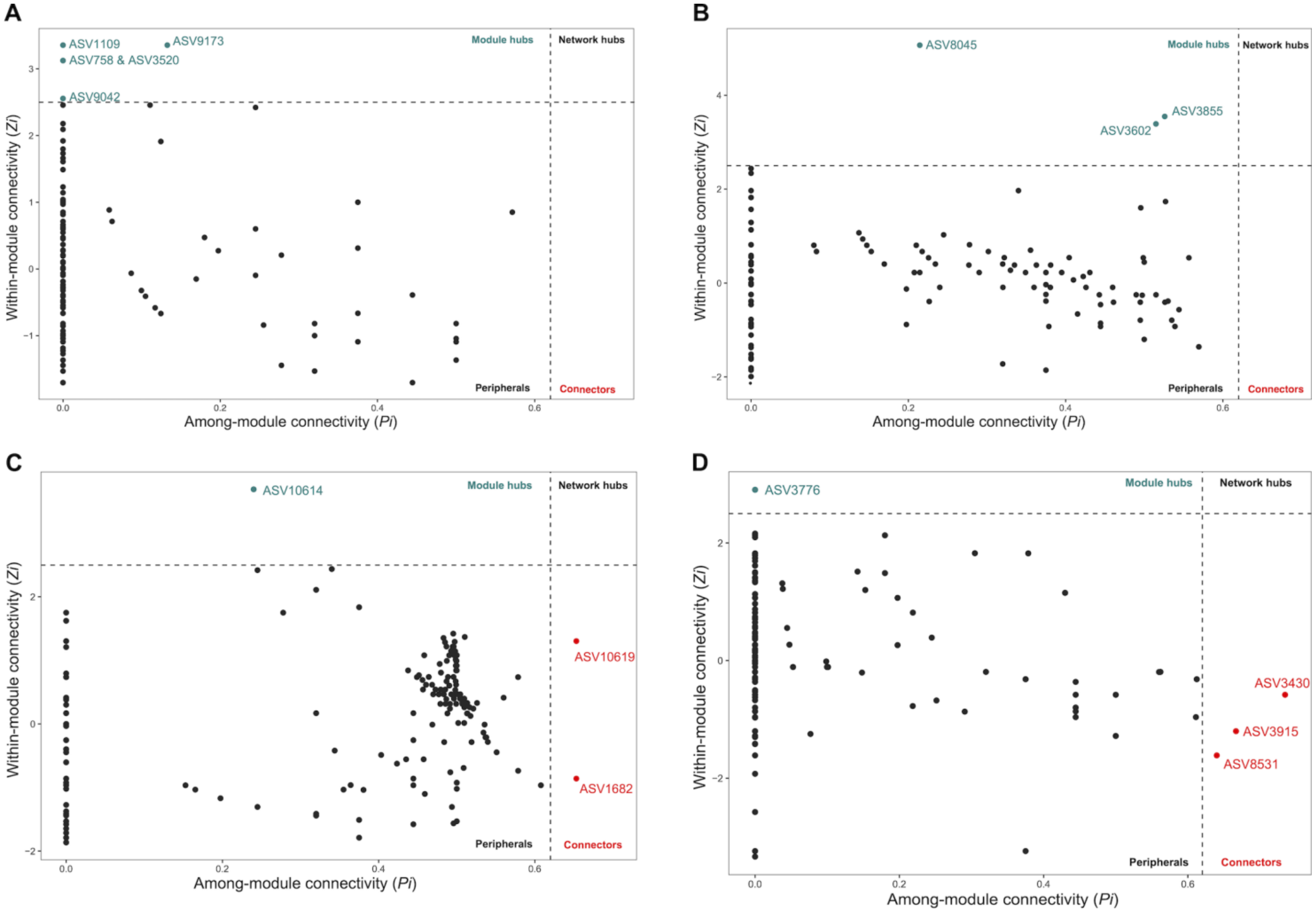

Fig. S7 **Keystone taxa of (A) PA, (B) ZA, (C) PF and (D) ZF bacterial communities identified based on among-module and within-module connectivity.** Module hubs and connectors are shown in green and red, respectively.
